# Oral lichen planus and thyroid gland diseases: possible associations

**DOI:** 10.1186/s12903-019-0859-5

**Published:** 2019-07-31

**Authors:** Lazar Kats, Yuli Goldman, Adrian Kahn, Victoria Goldman, Meir Gorsky

**Affiliations:** 1 Department of Oral Pathology, Oral Medicine and Maxillofacial Imaging, School of Dental Medicine, Tel Aviv University, 69978 Tel Aviv, Israel; 2 Department of Oral and Maxillofacial Surgery, School of Dental Medicine, Tel Aviv University, Tel Aviv, Israel; 3 Private practice, Holon, Israel

**Keywords:** Auto-immune disease, Oral lichen planus, Thyroid gland diseases

## Abstract

**Background:**

Lichen planus (LP) is a chronic inflammatory mucocutaneous disease that commonly affects the oral cavity. Previous reports have suggested a possible association between LP and thyroid gland diseases (TGDs). The purpose of this study was to investigate possible associations between oral lichen planus (OLP) and TGDs.

**Methods:**

Patients diagnosed with OLP, both clinically and histopathologically (*N* = 102), were classified according to clinical course (symptomatic/asymptomatic), type (reticular/plaque, atrophic and erosive) and location of lesions. Data on TGDs was compared to age- and gender-matched controls (*N* = 102) without OLP. Diagnosis of any type of TGD and related medication for study and control groups was recorded from the medical files provided by patients’ physicians. Statistical analysis used Student’s t-test and Fisher’s exact test; significance was set at *p* < 0.05.

**Results:**

TGDs (all), hypothyroidism and related medications were found in 16.6, 12.7 and 12.7% of patients with OLP, respectively. These findings were similar to the control group: TGDs (all) -15.7%, hypothyroidism - 9.8% and thyroid gland disease-related medication - 9.8% (*p* > 0.05). No significant associations were found between different characteristics of OLP and hypothyroidism or other TGD (*p* > 0.05).

**Conclusions:**

We found no significant associations between the co-existence of OLP and TGD or related-medications. Our findings are in agreement with some of the previously published similar studies but in controversy with others. Further well-designed, multicenter studies with large groups of patients and controls may help to establish the nature of the associations between OLP and TGDs.

## Background

Oral lichen planus (OLP) is a chronic immune-related condition of the oral mucosa of unknown exact etiology [1]. Current data suggest a prevalence of 1.27–2.0% in the general population [2]. Although OLP is considered as the oral manifestation or oral counter part of cutaneous LP, there are essential differences in the biological behavior of these two conditions, especially regarding the protracted course and possible association with malignant transformation of OLP. A diagnosis of OLP with no dermal manifestation is quite popular in the clinical setting of oral medicine with only 20% of the OLP patients having cutaneous LP [1].

The immune dysregulation is probably associated with T cell- and cytokine-mediated mechanisms [1–3], but mechanisms of humoral auto-immunity were also suggested [4]. The incapability to define the exact etiology of OLP can raise the possibility that OLP represents an immune response to different and varying causative triggers in inherently susceptible individuals [5]. Irrespective of the etiology, three main clinical forms of OLP are well recognized, reticular/keratotic, atrophic, and erosive. They differ in their appearance, symptomatology, management and prognosis [6, 7].

A possible association between OLP and thyroid gland diseases (TGDs), especially hypothyroidism, has been reported [8–10], which can be partially supported by the observation that several autoimmune conditions tend to be “clustered” with autoimmune TGDs. Since OLP is considered by some as an immune-mediated disease, it is reasonable to assume that it may be associated with other immune-mediated diseases, including autoimmune TGDs [10–12]. The prevalence of TGDs in OLP patients ranges between 6.2 to 15.3% compared to a lower prevalence of 1.6–8% in control groups, with odds ratio ranging between 1.47 and 3.01 [13]. A more recent investigation reported a prevalence of 72.4% of TGDs in OLP patients and of 49.4% in controls [14]. An increased level of auto-antibodies to the thyroid gland in OLP patients was also reported [12, 15, 16]. Furthermore, an association was found between OLP patients treated with Levothyroxine Sodium due to a diagnosis of hypothyroidism and the clinical course of OLP [17, 18].

In view of the wide range of reported prevalence of TGDs among OLP patients, we further add our experience and discuss updated insights in the immunologic etiology of TGDs.

## Methods

This is a retrospective case-control study that has been independently reviewed and approved by the Institutional Review Board of Tel-Aviv University, July 2012.

The study group included 102 patients who have been diagnosed, clinically and histopathologically, with OLP. All OLP patients were diagnosed and managed in the Oral Medicine Clinic of Tel Aviv University, between the years 2002–2012. The final diagnosis of OLP was based on diagnostic criteria proposed by vander Meij and colleagues [19]. Patients with a diagnosis of oral lichenoid lesions were excluded from the study. The findings of the study group were compared to 102 age- and gender-matched control patients who had no OLP. The control group included consecutive patients referred to the same clinic by dentists from the community during the same period of time. The control patients were managed for other oral findings, such as irritation fibromas, papillomas and fissured tongue. The anamnesis of all patients (both study and control groups) included a full report from the family physician comprising all systemic diagnoses and current medications.

OLP lesions were classified according to the clinical course, type and location. The clinical characterization was made based on the symptomatology: asymptomatic or symptomatic. OLP type was classified as reticular/plaque, atrophic and erosive forms. In case of a combination of different types, the lesions were defined based on the most severe clinical appearance.

Based on the information supplied by the family physician regarding the diagnosis of TGD and related medications, patients with TGDs were divided into two main groups, namely hypothyroidism and all other TGDs. TGDs were diagnosed based on routine studies of blood tests, ultrasound and when needed biopsy procedures. All additional relevant data, including concomitant systemic diseases/conditions and habits, like smoking and alcohol consumption, were also obtained from the medical files of the patients.

Statistical analysis was performed using the SPSS software (Chicago, USA), version 17. Comparison of parameters of age and gender between the OLP and control groups was performed by T-test. Associations between OLP and TGD (i.e., hypothyroidism, TGD-related drugs) were analyzed by Fisher’s exact test. Statistical significance was set at *p* < 0.05.

## Results

One hundred twenty-five medical files of OLP patients were initially examined, out of which only 102 fulfilled the diagnosis of OLP criteria. Females predominated both study and control groups (70.6%). The mean age at diagnosis in the OLP group was 55.7 + 13.2 years and that of the control group was 54.1 + 14.6 years, with no significant difference (*p* = 0.393).

The most common type of OLP was the reticular (54.9%), followed by erosive (27.5%) and atrophic (17.6%) types. Although the clinical manifestation of OLP was noted at any site of the oral cavity, the most commonly involved was the buccal mucosa (87.6%). Other sites included the gingivae (63.8%), tongue (40.2%), floor of the mouth (7.8%), palate (6.9%) and lip mucosa (5.9%). Symptoms were recorded in 53.9% of the OLP patients. Skin involvement was found in only 6.9%.

The most common TGD was hypothyroidism (Table 1). A medical history of hypothyroidism was found in 12.7% subjects of the OLP group compared to 9.8% of those in the control group (*p* = 0.659). No significant differences were found in the frequency of other TGDs, such as Hashimoto’s thyroiditis, between OLP group and the control group (*p* = 0.748). For all TGDs, the frequency was similar in OLP (16.6%) and control (15.7%) groups (*p* > 0.05). In addition, no differences were noted between these groups in regard to other co-existence of systemic conditions. Interestingly, smoking habit was significantly less common in the OLP group (11.8%) than in the control (24.5%) (*p* = 0.028); no statistically significant differences were found between groups regarding alcohol abuse (*p* = 0.621).

**Table 1:** Thyroid gland diseases, other systemic conditions and habits in the OLP and control groups (*N* = 102, each)

Characteristics	OLP Group	Control group	*P* value
Thyroid gland diseases			
Hypothyroidism	13 (12.7%)	10 (9.8%)	0.659
Others thyroid gland conditions	4 (3.9%)	6 (5.9%)	0.748
Thyroid gland disease-related medication			
Levothyroxine Sodium	13 (12.7%)	10 (9.8%)	0.659
Other concomitant systemic diseases			
Allergic conditions	15 (14.7%)	19 (18.6%)	0.574
Diabetes Mellitus	4 (3.9%)	5 (4.9%)	1
Dyslipidemia	21 (20.6%)	30 (29.4%)	0.196
Habits			
Smoking	12 (11.8%)	25 (24.5%)	**0.028**
Alcohol	3 (2.9%)	1 (1.0%)	0.621

Value in bold indicates statistical significance

Characteristics of OLP, including symptomatology, clinical type (reticular, atrophic and erosive) and oral sites were analyzed for associations with the diagnosis of hypothyroidism (Table 2). No significant associations (*p* > 0.05) were found. The same characteristics of OLP were also investigated for associations with the diagnoses of other TGDs. These also yielded no statistically significant results (p > 0.05).

**Table 2:** Associations between characteristics of OLP and thyroid gland diseases

OLP characteristics (N)	Hypothyroidism (N)	*P* value	Others Thyroid gland diseases (N)	*P* value
Symptoms (55)	6	0.567	2	1.000
OLP type				
Reticular OLP (56)	8	0.768	2	1.000
Atrophic OLP (18)	3	0.696	0	1.000
Erosive OLP (28)	2	0.506	2	0.302
OLP sites of lesions				
Tongue (41)	4	0.554	0	0.147
Buccal mucosa (89)	12	1.000	4	1.000
Gingiva (65)	9	0.765	3	1.000
Palate (7)	2	0.218	1	0.251
Floor of mouth (8)	0	0.592	0	1.000

## Discussion

The present study aimed to investigate any possible association between OLP and TGDs based on data documented in an academic dental center. Diagnosis of OLP was supported both clinically and microscopically in all cases. Diagnosis of TGDs was always confirmed by the patients’ records of the Health Maintenance Organization, to which each regular Israeli citizen is registered. In the current study we found that the prevalence of TGDs (all types and specifically hypothyroidism) in OLP patients was not significantly different from that of an age- and gender-matched control group (16.6% versus 15.7% for all TGDs and 12.7% versus 9.8% for hypothyroidism, *p* > 0.05). In addition, no significant associations were found between the clinical characteristics of OLP and TGDs. Interestingly, we found that smoking habit was significantly less common in the OLP group (11.8%) than in control (24.5%).

The relationship between OLP and TGDs has been investigated in a large number of studies from different parts of the world, yet the results were controversial. Lack of uniformity in study design and methodology can provide a partial explanation to this controversy. Patients with OLP were not always diagnosed by a specialist in oral medicine and their oral findings were usually not confirmed by histopathological examination, which was limited only to cases of an equivocal clinical impression. There were only a few studies, including the current one, in which it was clearly mentioned whether specialists in oral medicine have been involved in the diagnosis of OLP and whether both clinical and histopathological exams were performed [8, 10, 12]. The selected samples of OLP patients differed in the inclusion and exclusion criteria regarding OLLs, smoking habits and alcohol consumption [8, 10, 20, 21]. Diagnosis of TGDs sometimes relied on patients’ self-reporting data [9, 10, 20].

In general, auto-immune diseases of the thyroid gland show differential geographic prevalence. Hashimoto thyroiditis has an assessed prevalence of 300–2,980 per 100,000 in Europe-North America-Australia-New Zeeland zone (zone 1) and only 350 in Asia-Middle East-Caribbean-South America zone (zone 2) [22]. Likewise, OLP is also geographically related, ranging from 0.38% in Malaysia, 0.5% in Japan, 1.74% in USA, 1.9% in Sweden, and up to 2.6% in India [23, 24]. In line with this, it would have been expected to find associations between OLP and TGDs in geographical areas where both conditions show a similar trend of frequency. However, on practical grounds, it seems that this geographically-related association may not be valid, as even in the same geographical area, contradicting reports were found [12, 25, 26]. In the present study, TGDs were found in 15.7% of the patients in the control group. In studies from Israel, the frequency of hypothyroidism/TGDs has been reported to range from 4.54% [27] to 12.59% [28] and to 14% [29], irrespective of the levels of iodine in drinking water [30]. This implies that different parameters (e.g., year of study performance, sample size, group age of included patients) could have an impact on the frequency of TGDs. Thus, associations between OLP and TGDs on a geographical basis seem to be inconclusive, so far.

Up to 15% of patients aged 65 years and over may have subclinical hypothyroidism (mild thyroid failure, as reflected by an elevated thyroid stimulating hormone (TSH) above 4.0 μ IU/mL and normal free T4 levels), with only few, if any, symptoms suggestive of hypothyroidism [31]. The rate of progression to overt hypothyroidism is assessed to be about 5% per year, based on a combination of an elevated level of TSH with the presence of thyroid autoantibodies, thyroid peroxidase (TPO) and anti-thyroglobulin (Tg), together with clinical signs and symptoms, such as fatigue, weight gain, increased sensitivity to cold, drowsiness and others [22, 31]. Although the mean age of patients at time of diagnosis of OLP ranges between 36.9 years and 56.7 years [21], it is still younger than the estimated mean age when subclinical dysfunction in the thyroid gland begins. Since in most of the studies that attempted to show an association between OLP and TGDs there was no possibility to demonstrate a temporal link or sequence between the onset of OLP lesions and the abnormal thyroid-related levels of TSH, T4 and auto-antibodies, it becomes difficult to support such an association. Moreover, there is one study conducted in patients with auto-immune TGDs, which examined also involvement of OLP [32]. In that study, only one patient out of 65 (3%) was diagnosed with both TGD and OLP, which is substantially lower than that expected rate reported in studies that have investigated the frequency of TGDs among patients with OLP.

Some studies that investigated the association between OLP and TGDs also referred to the levels of thyroid gland hormones or thyroid auto-autoantibodies. In part of these studies, it was found that 84 to 95% of OLP patients had normal TSH levels [11, 16], with no difference when compared to control patients [11]. In contrast, a recent study has reported that a significant number of patients with OLP without diagnosed TGDs, showed thyroid parameters (e.g., high levels of TSH, low levels of free T4 and expression of TSH receptor in lesions of OLP) compatible with hypothyroidism [33]. In this regard, it should be mentioned that the levels of TSH may be suppressed by a number of medications such as steroids, dopamine, dobutamine, and octreotide [31]. Since OLP is treated by steroids, local or systemic, depending on the clinical severity of the disease [34], the precise TSH level in OLP patients might be concealed, thus making it difficult to conclude on any possible association between OLP and TGDs based on TSH levels. In addition, levels of auto-antibodies (i.e., anti-TPO and anti-Tg), which are considered more reliable in defining auto-immune TGDs, are known to be expressed in other medical conditions that are not related to thyroid diseases, such as asthma, idiopathic urticaria, rheumatoid arthritis, diabetes mellitus type 1 and others [22]. Furthermore, 10–15% of patients with Hashimoto thyroiditis may be serum antibody negative, while 10% of healthy young subjects and 15% of people > 60 years of age may have circulating anti-Tg [22, 31]. Exogenous factors, such as smoking and alcohol consumption have a protective effect with lowering the risk for TGDs. More specifically, smoking lowers the levels of anti-TPO and anti-Tg [22]. In line with this, the present study as well as another paper [8] reported a lower frequency of smokers among OLP patients compared to controls. In that paper the frequency of alcohol consumers was also lower in OLP patients compared to controls [8], which again, may influence the establishment of a possible association between OLP and TGDs.

## Conclusion

We have shown no association between OLP patients and TGDs in a group of OLP patients with well-documented medical data and clinical assessment performed by specialists in oral medicine. The present study agrees with some previous similar studies and contradicts the findings of others, further emphasizing the unresolved issue of a possible association between OLP and TGDs. Associations between OLP and TGDs seems to be complex and multifactorial, probably influenced by both endogenous and exogenous factors, some protecting and others promoting their inter-relations. It is necessary to establish multi-centric collaborations with larger study groups and increased inter-disciplinary collaborations between the field of oral medicine with those of endocrinology and epidemiology in an attempt to provide accurate characterization of the associations between OLP and TGDs.

## Data Availability

The datasets used and/or analyzed during the current study are available from the corresponding author on reasonable request.

## Abbreviations

LP: Lichen Planus
OLP: Oral Lichen Planus
Tg: Thyroglobulin
TGDs: Thyroid Gland Diseases
TPO: Thyroid Peroxidase
TSH: Thyroid Stimulating Hormone
